# What Is New in Classification, Diagnosis and Management of Chronic Musculoskeletal Pain: A Narrative Review

**DOI:** 10.3389/fpain.2022.937004

**Published:** 2022-07-15

**Authors:** Jiejie Zhuang, Houlian Mei, Fang Fang, Xiaqing Ma

**Affiliations:** ^1^Department of Anesthesiology, Suzhou Hospital of Anhui Medical University, Suzhou, China; ^2^Department of Nursing, Shanghai General Hospital, Shanghai Jiao Tong University, Shanghai, China; ^3^Kangda College of Nanjing Medical University, LianYunGang, China; ^4^Department of Anesthesiology, Affiliated Hospital of Nantong University, Medical School of Nantong University, Nantong, China

**Keywords:** musculoskeletal pain, classification, diagnosis, management, advances

## Abstract

Chronic musculoskeletal pain (CMP) is the most common type of chronic pain, defined as persistent or recurrent pain condition deriving from musculoskeletal structures such as muscles, joints or bones that lasts for more than 3 months. CMP is multifactorial and severely affects people's quality of life. CMP may be influenced by a number of factors, including contextual factors, the presence of comorbidities, arthritis coping efficacy and access to CMP care. To deepen the comprehensive understanding of CMP, this narrative review provides the latest literature on disease classification, clinical diagnosis, treatment and basic research. In terms of the classification of the disease, here we introduce the 11th edition of the International Classification of Diseases (IDC-11), in which CMP is divided into chronic primary musculoskeletal pain and chronic secondary musculoskeletal pain. In the clinical diagnosis section, the progress of central sensitization in the diagnosis of CMP will also be summarized. In addition, we summarize some recent advances in clinical treatment and basic research.

## Introduction

CMP involves more than 150 diseases of the human motor system, including common chronic joint pain, neck and shoulder pain, low back pain, limb pain, spinal pain, fibromyalgia and myofasciitis. Musculoskeletal disorders affect 20 to 33% of the global population. Although the prevalence of CMP reported in various literatures is inconsistent, it is certain that it increases with age, among which low back pain is the most common, with an incidence of 30–40% in adults. A recent report in the United States indicates that one in two adults has CMP, which is equivalent to the incidence of cardiovascular disease and chronic respiratory disease combined.

Despite the prevalence and impact of CMP, as well as the proliferation of new therapies, CMP is still a major challenge to clinical and basic research. There are many kinds of CMPs, and the pathogenesis of different diseases if different, which may involve one or more of inflammatory responses, fibrosis, release of neurotransmitters, abnormal changes in neuroimmunity, peripheral sensitization and central sensitization.

Pain is the most common symptom of CMP and the most critical factor in the burden of the disease. Chronic pain not only seriously affect the daily life of patients, but tends to develop into depression, which further causes the refractory CMP. Therefore, in order to better treat CMP, it is not only necessary to understand the specific etiology of the disease, to treat the cause of the disease, but also to carry out corresponding symptomatic treatment, whether physical or psychological symptoms. The newly released IDC-11 divided CMP into primary and secondary, which provided a new direction for better understanding of the etiology, diagnosis and treatment of CMP.

## The Classification of Chronic Musculoskeletal Pain

In the 10th edition of the International Classification of Diseases (ICD-10), symptoms associated with known pathological conditions affecting the musculoskeletal system or connective tissue or unknown pathologies at specific locations are collectively referred to as musculoskeletal pain. The former is common as osteoarthritis (OA), autoimmune inflammatory arthritis (RA). “Back pain” or “periarticular pain” fall into the more common category of the latter. According to the ICD-10, pain with a known cause is assigned to the diagnostic code for the disease, not the pain code. However, for patients who seek help primarily to relieve pain rather than treat an underlying condition, a pain code would ideally be assigned. Whereas, in ICD-10, composite code is not part of the coding strategy.

The ICD-11 split CMPs into two main types: chronic primary musculoskeletal pain and chronic secondary musculoskeletal pain (1). Chronic primary musculoskeletal pain is defined as chronic pain experienced in muscles, bones, joints, or tendons comorbid with significant emotional distress or functional disability, and cannot be attributed directly to a known disease or damage process, such as chronic non-specific low back pain and chronic widespread pain.

Chronic secondary musculoskeletal pain is a kind of CMP arising from a known disease and is commonly due to several main causes: ① persistent local or systemic inflammatory diseases caused by crystal deposition, infection, autoimmune or autoinflammatory processes; ② local musculoskeletal structural changes; ③ musculoskeletal problems caused by neurological diseases, such as muscular hypertonicity in Parkinson disease.

This new classification combines the underlying mechanisms of chronic pain with early musculoskeletal disorders to provide a more accurate epidemiological analyses of diseases characterized by musculoskeletal pain. The change in classification will allow for patient-centered management, not just pathophysiology driven by disease, and will mean the need for multimodal treatment of chronic pain.

## The Assessment of Musculoskeletal Pain

Diagnosis of CMP requires a detailed history, careful physical examination, and careful laboratory and imaging tests to rule out infections, tumors, and other diseases. What's more, once CMP is diagnosed, it is also important to assess the pain and related conditions, which is conducive to the diagnosis of the disease and the determination of treatment plan. Since the occurrence of CMP is believed to be closely related to the occurrence of central sensitization, the progress of central sensitization in the diagnosis of CMP will also be summarized here.

### The Assessment of the Pain Intensity

The most commonly used assessment tools for pain intensity include the Numerical Rating Scales (NRS), Verbal Rating Scales (VRS), Visual Analog Scales (VAS), and the Faces Pain Scale-Revised (FPS-R) (2, 3). In clinical, we can choose an appropriate assessment method according to the specific situation of the patient. NRS is a method for patients to describe their pain intensity on a scale of 0–10, which is more suitable for patients with less education and understanding. When assessed using VRS, patients are asked to choose from a list of words (no pain, mild pain, moderate pain, severe pain, extreme pain) that better describe their pain intensity. This method is easy to understand, but it is more suitable for history-taking and follow-up because different patients have different comprehension for adjectives. When using the VAS method, a 10 cm ruler should be prepared in advance, with the 0 cm end representing no pain and the 10 cm end representing unbearable severe pain, and then patients should choose a length representing their pain intensity. This is also a common evaluation method in clinical practice. But the results may vary greatly with patient's cognitive ability, level of education and comprehension. For patients such as elderly, infants and other groups, who cannot evaluate the intensity of pain with NRS, VRS or VSA, we can judge by some pain-related behavioral changes. FPS-R is the commonly used method, which is especially suitable for children.

Another common way to assess the pain intensity is through questionnaires, and the McGill Pain Questionnaire is the most classic and comprehensive questionnaire used in clinical practice (4). The questionnaire involves not only the intensity of the pain, but also the character of the pain. It contains a number of words describing pain that are grouped into four categories: feeling, emotion, evaluation and miscellaneous.

### Contextual Factors and Psychological Assessment

As with other chronic pain conditions, several key contextual factors can influence pain perception during CMP. These include the presence of comorbid health problems (5, 6), social support (7, 8), sex/gender (9), education and heath literacy, income, personality. This explains why people with radiologically similar arthritis severity may experience varying degree of pain or other chief complaint experience (9). Thus, CMP assessment requires a bio-psychosocial perspective including not only the assessment of pain, but also its downstream effects and contextual factors. Clinically, GAD-7, PHQ-9 and PHQ-15 scales are commonly used to evaluate the existence and degree of psychological disorders in patients (10, 11).

### Function Assessment

The Short Form 36 (SF-36) is commonly used to evaluate the function of CMP patients. The SF-36 is a self-administered universal measure of health-related quality of life (QoL) that focuses on both physical and mental aspects. It includes 36 projects in eight health areas. The total Likert scale was used in this health survey. The scores are eventually translated to 0–100, with a higher score representing a better health-related QoL.

In trials of OA, the Western Ontario and McMaster Universities Osteoarthritis Index (WOMAC) is the most commonly used outcome measure to evaluate symptoms (12). Another questionnaire often used is the Nordic Standardized Musculoskeletal Symptoms Questionnaire (NMQ) (13).

### The Assessment of Central Sensitization in CMP

A distinct feature of CMP is central sensitization, which is defined as increased reactivity of nociceptive neurons, such as increased excitability, enhanced synaptic transmission, and reduced inhibition in the central nervous system (CNS) to normal input. So far, there are no objective indicators of central sensitization *in vivo*, but some signs and symptoms may be suggestive in clinical practice.

Quantitative sensory testing (QST) is a clinical surrogate marker for central sensitization that comprehensively assesses sensitivity to a range of stimuli, especially enhanced reactivity (14). QST is a promising method for detecting central sensitization, but it could only evaluate evoked responses, not spontaneous pain. The successful use of QST relies primarily on testing skin sensitivity, rather than deep pain arising from deep tissues. Dynamic mechanical ectopic allodynia can be examined by brushing the skin. This could be a manifestation of central sensitization, but may also be caused by peripheral drives (15). Further research is needed to apply objective markers of central sensitization, such as biomarkers and functional neuroimaging, into clinical practice.

## The Treatment of Chronic Musculoskeletal Pain

CMP is still a huge challenge for both clinicians and researchers. It is widely believed that the burden of CMP will continue to increase (16) as populations age and the global obesity epidemic increases. The main objectives of CMP treatment are to relieve pain, improve function and improve quality of life. The chronic primary musculoskeletal pain is more common in primary care, but as management progresses, many are subclassified as chronic secondary musculoskeletal pain. Chronic secondary musculoskeletal pain is a kind of CMP arising from a known disease and is commonly due to several main causes, so the treatment can be more inclined to the etiological treatment. At present, in clinical practice, the mainstream treatment method is the combination of pharmacological and non-pharmacological approaches, and assisted by exercise, weight reduction and education of multi-mode treatment (17). Analgesic or surgical interventions are recommended (18, 19) based on disease severity and joint site.

### Pharmacological Therapy for CMPs

There are many options available for treatment of CMP, however, pharmacological remains the mainstay. This part will provide an overview for clinicians of recent advances in knowledge on the use of existing pharmacological therapies for CMPs.

#### Non-opioid Analgesics Therapy for CMPs

##### NSAIDs

NSAIDs are a class of non-steroidal drugs that have antipyretic and analgesic effects by inhibiting the activity of cyclooxygenase (COX) and then reducing the synthesis of prostaglandins (20). Although NSAIDs have a good analgesic effect on mild to moderate CMP caused by inflammatory factors, their therapeutic effect also has a ceiling effect. Therefore, increasing dose and the combined use of two NSAIDs drugs should be avoided in clinical use.

NSAIDs can be classified into non-selective COX inhibitors and selective COX-2 inhibitors according to their selectivity for cyclooxygenase subtypes. The two classes of NSAIDs have their own adverse reactions. Non-selective COX inhibitors are more harmful to gastrointestinal mucosa, while selective COX-2 inhibitors are more likely to cause cardiovascular and renal adverse reactions (21). Therefore, NSAIDs should be used with caution or avoided in patients with a history of upper gastrointestinal ulcer, kidney disease or ischemic heart disease.

#### Cannabis-Based Medicines

It has been speculated that dysfunction of the endocannabinoid system may be one of the causes of persistent pain in osteoarthritis (22–24). As traditional treatments for CMPs are imperfect, treatment options such as cannabis-based medicines may hold promise.

Studies show that the most common reason that patients are using medical cannabis (MC) if for management CMP (25). Chronic low back pain, as a common musculoskeletal disorder, is one of the most common reasons patients consult in primary care. Fearful of the many side effects of opioid use, many patients may turn to MC for effect. However, evidence on MC as a treatment of CMP is still lacking.

An 8-week cross-over RCT study of spinal pain and headache showed that the intensity of spinal pain was significantly lower in the nabilone group than in the placebo group. Moreover, participants generally preferred nabilone to placebo during the medication switch period (26). In a retrospective cohort study of 61 patients who use prescription opioids, almost half of the patients think of the use of MC can reduce the use of prescription opioids, however, also found at the same time, the only way to stop the use of opioids is higher doses of cannabinoid, with the adverse event also increases (27).

Although MC has been shown to be effective in many chronic pain models, risks should be weighed against those of other current treatments, such as opioids.

#### Serotonin-Noradrenaline Reuptake Inhibitor (SNRIs)

SNRIs, represented by duloxetine and venlafaxine, are commonly used clinically as a class of antidepressant drugs. Studies have shown that SNRIs have significant analgesic and antidepressant effects on various kinds of chronic musculoskeletal pain (28, 29). The mechanism of SNRIs alleviating CMP pain mainly involves enhancing the role of the descending inhibitory system and reducing the pain stimulation signals transmitted through the spinal cord.

The 2014 Guidelines for the Treatment of Knee Osteoarthritis issued by the Osteoarthritis Research Society International (OARSI) are the first to include duloxetine as a recommended treatment. Studies have shown that duloxetine is more effective in combination with NSAIDs and has additional benefits for patients with concurrent depressive symptoms (30, 31). Other studies have shown that duloxetine has a direct analgesic effect in the treatment of pain, rather than just antidepressant effect (32). The cause of fibromyalgia, which is characterized by widespread pain in muscles and soft tissues throughout the body, remains unknown. Studies have shown that duloxetine can significantly reduce the pain scores of patients with fibromyalgia, regardless of depression (33).

Compared with NSAIDs, SNRIs has a good safety profile in gastrointestinal and cardiovascular adverse events, but there are some unavoidable side effects such as nausea, dizziness, constipation, and loss of appetite.

#### Muscle Relaxant

Muscle relaxants used in clinical practice can be classified into two types: skeletal muscle relaxant (baclofen, dantrolene) and central muscle relaxant (benzodiazepines, non-benzodiazepines, tizanidine).

#### Ion Channel Drugs

Clinical ion channel agents include sodium channel blockers (such as carbamazepine, oxcarbazepine, lidocaine, etc.), calcium channel modulators (such as gabapentin, pregabalin) and potassium channel openers [flupirtine, have been withdrawn from the market due to its hepatotoxicity (34)]. Calcium channel modulators are most widely used in CMP. The main mechanisms by which calcium channel blocker relieve pain are: inhibition of calcium influx and reduction of neurotransmitter release, thereby reducing abnormal excitation of pain transduction pathways (35).

#### Topical Drugs

Topical non-opioid analgesics for the treatment of CMP include NSAIDs, local anesthetics, capsaicin, and traditional Chinese medicines (TCMs). Topical drugs can directly penetrate into the affected tissue through the skin to play an analgesic effect. It has the advantages of fast acting, high local concentration, less systemic exposure and fewer adverse reactions. Compared with oral preparations, topical drugs are more suitable for long-term CMP management (33, 36, 37).

#### Opioid Therapy for CMPs

As one of the leading causes of disability, osteoarthritis affects more than 500 million people worldwide (38). Basic treatments for OA include exercise and maintaining a healthy weight, as well as analgesics based on NSAIDS and acetaminophen. However, the role of opioid analgesics in the treatment of OA varies from country to country (37, 38). In the United States, approximately 40% of patients with knee osteoarthritis use opioids (39).

A recent review evaluated the efficacy and safety of opioid analgesic regimens in patients with osteoarthritis. The researchers found that the pain and disability benefits of opioids in patients with osteoarthritis were minimal, but they may increase the risk of adverse events (40).

### Non-pharmacological Management of CMPs

#### Acupuncture Treatment for CMPs

Available evidence indicates that acupuncture may be a safe option for providing short-term pain relief in the treatment of knee osteoarthritis (41, 42) and chronic low back pain (43). Other studies have shown that acupuncture is also beneficial for fibromyalgia (44). However, there is a lack of positive evidence regarding the use of acupuncture in osteoarthritis of the hip and rheumatoid arthritis (45).

#### Local Anesthetic Injection for CMPs

Local anesthesia options for the treatment of CMP include paracetamol (acetaminophen) and intraarticular corticosteroid injections if pain is moderate to severe (17). However, the guidelines indicate that the clinical and economic evidence for intra-articular corticosteroid injections is limited and that the available evidence is inconsistent (17). Additional results suggest that intraarticular corticosteroid injections may be used in osteoarthritis of the knee, but not in osteoarthritis of the hip (46).

### Recommendations for Lifestyle Improvements in People With CMPs

Physical activity (including exercise) is the cornerstone of the treatment of musculoskeletal pain (47, 48). To evaluate the beneficial of physical activity on strength, flexibility and cardiovascular fitness in people with rheumatoid arthritis, spondyloarthritis and hip/knee osteoarthritis, a systematic review and meta-analysis were performed in 2018. According to the systematic review, the European League Against Rheumatism (EULAR) recommended physical activity for people with inflammatory arthritis and OA (49). Unfortunately, little is known about the best dose and type of exercise, especially whether exercise is better with or without pain. A recent systematic review of pain-exercise vs. pain-free exercise for CMP found that a pain-exercise regimen had a small but statistically significant short-term benefit over pain-free exercise (50). When exercise is painful, the immune system and the affective aspects of pain may provide some additional benefits. These mechanisms can alleviate and relieve musculoskeletal pain and, with appropriate clinical support and education, redefine pain as safe, non-threatening exercise. In addition, allowing painful exercise can lead to greater load/amount of movement, thus promoting functional recovery.

Since weight load is closely related to exercise, weight loss and obesity should be considered as a strategy to benefit global health, especially for CPM patients. The data from over 400 published reviews and original articles suggests that across he seven musculoskeletal diseases (osteoarthritis, rheumatoid arthritis, systemic lupus erythematosus, axial spondyloarthritis, psoriatic arthritis, systemic sclerosis and gout), moderate-quality evidence suggested that he heavier a patient was, the overall prognosis, including pain, function and activity, was worse (51).

In conclusion, given the beneficial effects of exercise on numerous outcomes, sufficient amounts of exercise are recommended. Furthermore, Also, a healthy lifestyle and an appropriate weight are recommended to avoid the negative consequences of being overweight.

### Multimodal Chronic Pain Therapy for CMP

Due to the COVID-19 pandemic, many CMP patients are unable to see their doctors regularly in person. In response to this situation, a randomized controlled clinical trial has been conducted, which shows that app-based mobile multidimensional therapy improved catastrophizing, quality of life, and mental flexibility immediately after treatment in adults with CMP, and the main outcome of catastrophizing remained effective at least 3 months following treatment. In addition, they can promote self-management and can be used to complement face-to-face pain treatments (52).

Another interesting exploratory randomized controlled trial was to observe the preliminary effectiveness of an immersive virtual reality (VR) multimodal therapy for older adults with chronic back pain (CBP) in a laboratory setting over a period of four weeks. It showed that only a significant improvement in the subjective functional capacity after the completion of a four-week multimodal pain therapy in VR. There were no significant differences in ear-avoidance beliefs and general physical and mental health. Although VR therapy did not achieve the reduction in pain intensity achieved by traditional multimodal pain therapy, the results of this study suggest that current VR therapy can achieve the reduction in pain intensity. In general, it would be considered as an adjunctive therapy for multimodal pain management, but cannot be used as a substitute in its current prototype (53).

## From Basic Research to Clinical Administration

CMP is a multifactorial disease and not all patients with CMP have the same symptoms or rate of disease progression. This has led to disagreement (or lack of consensus) in the community about the drivers of CMP progression and pain. However, to date, it has been thought to involve a number of components stemming from central processing dysfunction, including central sensitization and impaired processing in the descending inhibitory pain pathway.

The changes of cortisol levels in patients with fibromyalgia (FM) are inconsistent in different researches. But the lower levels of cortisol are thought to be due to a maladaptive response to stress resulting from a central abnormality of the hypothalamic-pituitary-adrenal axis (54). The endocannabinoid system is also reported to be altered in animal models of FM. Hong et al. demonstrated a downregulation of CB1 receptor expression in the dorsal root ganglia, whereas, the levels of mRNA expression of AEA and TRPV1 receptors were significantly elevated (55, 56).

Animal models are a useful approach to unravel the complexity of CMPs. Using multiple models to validate a novel therapy or target will allow a better understanding of how these models relate to specific pathological changes in patients with CMP, and how novel therapies can mitigate or prevent these changes (57).

Tendinopathy is a common problem affecting active young and middle-aged people, which has a significant impact on their personal and professional activities (58), even leading to up to 53% of athletes with patellar tendinopathy had to quit their sports career because of knee problem (59). Results of a recent study in a large animal, piglets, showed that ultrasound-guided injection of type 1 collagenase inside the patellar tendon allowed the generation of a tendinopathy model with neovessels in piglets. In addition, the piglets that successfully modeled the pigs showed better tolerance and no suffering sign (60). The significance of this study not only suggests the feasibility of large animals such as piglets in basic research, but also predicts the potential role of type 1 collagenase in the treatment of tendinopathy.

However, there is still a huge gap between basic research and clinical application. One of the most obvious and long-standing differences is gender. As women are two-thirds more likely than men to develop osteoarthritis, and they report greater pain, it's better to use both male and female animals in preclinical OA studies so that sex-specific differences in OA pathogenesis and pain perception can be tease out. However, this point is not well done in current studies, and most basic experiments only use male animals as research objects for various reasons.

In addition, using multiple different methods to measure pain from all aspects in a particular model can assess multiple pain patterns, as well as changes in pain sensation and mechanisms over time. Only by using multiple animal models of OA to understand pain mechanisms can we hope to identify drug and non-drug targets to eliminate this debilitating disease.

## Conclusions

In summary, this narrative review briefly summarizes the new classification of diseases (ICD-11). Clinical diagnostic protocols were also summarized, including assessment of pain intensity, patient background and psychological status, functional status, and central sensitization. In addition, on the basis of summarizing the classical treatment options, we introduce the methods involved in the latest research, such as VR and mobile phone based multi-mode analgesia, which would be more suitable for the current COVID-19 epidemic.

## Author Contributions

XM and FF conceived the article. JZ and HM revised and reviewed the article. All authors contributed to the article and approved the submitted version.

## Conflict of Interest

The authors declare that the research was conducted in the absence of any commercial or financial relationships that could be construed as a potential conflict of interest.

## Publisher's Note

All claims expressed in this article are solely those of the authors and do not necessarily represent those of their affiliated organizations, or those of the publisher, the editors and the reviewers. Any product that may be evaluated in this article, or claim that may be made by its manufacturer, is not guaranteed or endorsed by the publisher.
